# Risk of Polycystic Ovary Syndrome in Women Exposed to Fine Air Pollutants and Acidic Gases: A Nationwide Cohort Analysis

**DOI:** 10.3390/ijerph16234816

**Published:** 2019-11-30

**Authors:** Shih-Yi Lin, Yu-Cih Yang, Cherry Yin-Yi Chang, Cheng-Chieh Lin, Wu-Huei Hsu, Shu-Woei Ju, Chung-Y. Hsu, Chia-Hung Kao

**Affiliations:** 1 Graduate Institute of Biomedical Sciences and School of Medicine, College of Medicine, China Medical University, No. 2, Yuh-Der Road, Taichung 40447, Taiwan; oasisbestonly@yahoo.com.tw (S.-Y.L.); D4754@mail.cmuh.org.tw (C.Y.-Y.C.); cclin@mail.cmuh.org.tw (C.-C.L.); hsuwh@mail.cmuh.org.tw (W.-H.H.); D14315@mail.cmuh.org.tw (S.-W.J.); hsucy63141@gmail.com (C.-Y.H.); 2 Division of Nephrology and Kidney Institute, China Medical University Hospital, Taichung 40402, Taiwan; 3 Management Office for Health Data, China Medical University Hospital, Taichung 40402, Taiwan; moiluvkiwi@gmail.com; 4 College of Medicine, China Medical University, Taichung 40402, Taiwan; 5 Department of Gynecology, China Medical University Hospital, Taichung 40402, Taiwan; 6 Department of Family Medicine, China Medical University Hospital, Taichung 40402, Taiwan; 7 Department of Chest Medicine, China Medical University Hospital, Taichung 40402, Taiwan; 8 Department of Nuclear Medicine and PET Center, China Medical University Hospital, Taichung 40402, Taiwan; 9 Department of Bioinformatics and Medical Engineering, Asia University, Taichung, 40402, Taiwan; 10 Center of Augmented Intelligence in Healthcare, China Medical University Hospital, Taichung, 40402, Taiwan

**Keywords:** air pollutants, polycystic ovary syndrome (PCOS), Taiwan air quality, monitoring database

## Abstract

Background: Air pollutants cause endocrine disorders and hormone disruption. The relationship between air pollutants and polycystic ovary syndrome (PCOS) must be carefully investigated using a nationwide cohort. Methods: Data were extracted from two nationwide databases, namely Longitudinal Health Insurance Database and Taiwan Air Quality Monitoring Database, and analyzed. The study considered a range of data that began on 1 January 2000 and ended on 31 December 2013. Women diagnosed with PCOS were excluded. From the residential data, the study assessed the daily concentrations of sulfur dioxide (SO_2_), nitrogen oxides (NOx), nitrogen monoxide (NO), nitrogen dioxide (NO_2_), and PM_2.5_ the women were exposed to. A Cox proportional hazard regression model was applied to assess PCOS risk. Results: In total, 91,803 women were enrolled in this study; of those women, 2072 developed PCOS after 12 years of follow-up. The mean daily concentrations of SO_2_, NOx, NO, NO_2_, and PM_2.5_ women were exposed to were 4.25 (±1.44) ppb, 20.41 (±6.65) ppb, 9.25 (±4.36) ppb, 20.99 (±3.33) ppb, and 30.85 (±6.16) μg/m^3^, respectively. Compared with the first-quartile levels of exposure, the fourth-quartile levels of exposure to SO_2_, NOx, NO, NO_2_, and PM_2.5_ increased PCOS risk by 10.31 times (95% CI = 8.35–12.7), 3.37 times (95% CI = 2.86–3.96), 4.18 times (95% CI = 3.57–4.89), 7.46 times (95% CI = 6.38–8.71), and 3.56 times (95% CI = 3.05–4.15), respectively. Conclusion: Women exposed to a high concentrations of air pollutants, namely SO_2_, NO, NO_2_, NOx, and PM_2.5_, had a high PCOS risk.

## 1. Introduction

Air pollution has drastically worsened in recent decades [1,2,3,4,5,6]. Air is essential for maintaining the biochemical activity of the cells within the human body [7]. Therefore, inhalation of polluted air could cause physiological hazards ranging from short-term cough, breathing difficulty, or headache [8] to long-term diseases including cancer [9], cardiovascular disease [8], dementia [10], mortality [11], and endocrine dysfunction [12]. It has been reported that global attributable deaths of air pollution increased by 89% to 124% over the period 1960 to 2009 [2]. Forouzanfar et al. identified exposure to ambient PM_2.5_ as a major contributing risk factor to disease [13].

The effects of air pollution on reproductive function were recently also investigated [14]. Selevan et al. showed that exposure to air pollutants may cause alterations in sperm quality in men [15]. The prospective cohort study conducted by Mahalingaiah et al. indicated that increased exposure of any maternal-aged woman to air pollutants may increase infertility risk [16]. A systemic review by Conforti et al. [17] showed that exposure to PM_2.5_, PM_2.5–10_, traffic pollutants, and sulfur dioxide (SO_2_) can increase the infertility rate and decrease the conception rate. Ciarrocca et al. [15] and Monti et al. [16] reported alterations in the levels of plasma follicular-stimulating hormone and luteinizing hormone in female outdoor workers exposed to air pollution. 

Furthermore, it has been reported that long term exposure to air pollutants causes inflammation [18]. Chung et al. reported that air pollution causes inflammation, oxidative stress, and autonomic dysfunction [18]. Sun et al. also found that air pollutants cause vascular inflammation in mice models [19]. Although the real mechanism of polycystic ovary syndrome (PCOS) remains unknown, it has been proposed that PCOS is associated with increased oxidative stress and inflammation [20,21,22]. It has also been reported that pro-inflammatory stimuli could upregulate the ovarian theca cell steroidogenic enzyme and thus cause an increase of androgen production, which might contribute to the development of PCOS [23]. Despite studies that have analyzed the effects of air pollution on fertility and hormonal levels, the association between polycystic ovary syndrome (PCOS) and air pollution has seldom been investigated. PCOS is associated with hormone deregulation and can be a risk for infertility [24]. 

Further, PCOS is also associated with an increased risk of type 2 diabetes and cardiovascular events [25,26] PCOS has been regarded as the most frequently encountered endocrinopathy in women and is associated with significant morbidity, including cancer [27]. Most previous studies have addressed the effect of environmental estrogen and bisphenol A (BPA) on PCOS development [28,29]. Nationwide cohort studies on the association between fine air particles and PCOS are lacking.

In addition, in recent years, air pollution in Taiwan has worsened because of increased coal combustion in the Taichung power plant in mid-Taiwan, one of the largest top ten coal-burning power plants in the world [30,31]. In another aspect, Taiwan has launched a national medical insurance with coverage over 95% residents, providing a suitable resource for a country-based study [32]. Therefore, it was meaningful to study incidence of PCOS in air-polluted Taiwan. We conducted a population based-cohort study to provide epidemiological information about the association between exposure to air pollutants and PCOS.

## 2. Methods

### 2.1. Data Source

We extracted data from the National Health Insurance Research Database (NHIRD) through the Longitudinal Health Insurance Database (LHID) and the Taiwan Air Quality Monitoring Database (TAQMD) to conduct this cohort study. One million Taiwan residents who participated in the National Health Insurance program were randomly selected, and their inpatient claims, outpatient claims, and personal basic information were recorded in the LHID. The identification data were encoded before making them available for research to protect the privacy of all patients. The International Classification of Diseases, Ninth Revision, Clinical Modification (ICD-9-CM) was used to identify the disease in the LHID. The TAQMD provided data from 78 air quality monitoring stations distributed across Taiwan. The daily concentration data of SO_2_, nitrogen oxides (NOx), nitrogen monoxide (NO), nitrogen dioxide (NO_2_), and suspended particulates (PM_2.5_) obtained from the TAQMD were used in this study.

### 2.2. Sample Participants

The study began on 1 January 2000 and ended on 31 December 2013. For each of the six municipalities (Taipei, New Taipei, Taoyuan, Taichung, Tainan, and Kaohsiung) (Supplementary Figure S1), women who lived in any of that municipality’s three most populous districts were enrolled into our study. Thus, the 18 selected administrative districts were Da’an District, Neihu District, and Shilin District of Taipei City; Banqiao District, Zhonghe District, and Xinzhuang District of New Taipei City; Taoyuan City, Zhongmu City, and Pingzhen District of Taoyuan; North District, Xitun District, and Taiping Township of Taichung City; East District, Annan District, and Yongkang District of Tainan City; and Zuoying District, Sanmin District, and Fengshan District of Kaohsiung City (Supplementary Figure S2). We excluded patients who were male or had polycystic ovary syndrome or who lacked continuous health insurance coverage preceding before entry. The index date of these study participants was 1 January 2000. We also adjusted confounding factors such as living area, urbanization level, monthly income, and occupational class. The urbanization level was categorized by the population density of the residential area into four levels, with level one as the most urbanized and level four as the last urbanized. The occupational class was categorized by kind of occupation into three types: white collar class included people who work in offices such as government officials or general employees; blue collar class included workers who work in areas needing strength or physical skill such as farmers or fishermen; other class included alternative military service, monks, or priests. A total of 91,803 female participants were included as study cohorts, and they were followed until the first diagnosis of polycystic ovary syndrome, National Health Insurance program withdrawal, death, or the end of the study.

#### Exposure Assessment

Due to the absence of evidence regarding the duration of exposure to air pollution that causes PCOS, we used data regarding exposure to air pollutants for one year before the diagnosis of PCOS. We estimated the daily concentrations of SO_2_, NOx, NO, NO_2_, and PM_2.5_ by using the inverse distance weighting (IDW) method. The IDW method is one of the most commonly used spatial interpolation methods in geosciences. The IDW method calculates the prediction values of unknown points by obtaining the averages of the values of known points. We subsequently integrated the daily concentrations of air pollutants corresponding with residential zip codes to calculate the annual average exposure to air pollutants (Supplementary Figures S3–S7). The concentrations of each air pollutant were divided into four quartiles: SO_2_: first quartile (Q1): <2.98 ppb, second quartile (Q2): 2.98–3.93 ppb, third quartile (Q3): 3.93–5.4 ppb, and fourth quartile (Q4): >5.4 ppb; NOx: Q1: <24.39 ppb, Q2: 24.39–30.32 ppb, Q3: 30.32–34.61 ppb, and Q4: >34.61 ppb; NO: Q1: <5.47 ppb, Q2: 5.47–7.21 ppb, Q3: 7.21–12.21 ppb, and Q4: >12.21 ppb; NO_2_: Q1: <19.05 ppb, Q2: 19.05–22.31 ppb, Q3: 22.31–22.95 ppb, and Q4: >22.95 ppb; PM_2.5_: Q1: <27.23 μg/m^3^, Q2: 27.23–27.41 μg/m^3^, Q3: 27.41–34.78 μg/m^3^, and Q4: >34.78 μg/m^3^. The variables of age, living area, urbanization level (categorized by the population density of the residential area into four levels, with level one as the most urbanized and level four as the least urbanized), monthly income, and occupational class (white-collar class, blue-collar class, and other) were considered confounding factors.

### 2.3. Statistical Analysis

Chi-squared testing was used to compare the differences in the urbanization level of each quartile of the daily average concentration of air pollutants. The incidence of PCOS (per 10,000 person-years) was calculated at each level of air pollutant concentration. Using the Cox proportional hazard regression model, the relative risk of PCOS among participants who were exposed to Q2–Q4 of air pollutant corresponding to those exposed to Q1 of air pollutant was estimated. Subsequently, a multivariate Cox proportional hazard regression model was then used to adjust for age, monthly income, urbanization level, and occupational class. According to the stratification of age, urbanization, monthly income, and occupational categories, the relative risk of developing PCOS in patients exposed to Q2–Q4 air pollutants in comparison with those exposed to Q1 air pollutants was analyzed.

## 3. Results

Table 1 shows the baseline characteristics and the air pollutant exposure of the study cohort. In total, 2072 participants developed PCOS after 12 years of follow-up. The mean follow-up time was 7.76 (±3.79) years. Approximately 70% of the participants were in age group 20–64 years, and the mean age of the study cohort was 36.9 (±18.8) years. Participants mainly lived in the north (65.7%) and the urbanization level two (51.6%) areas. Monthly income of most of the participants (56.9%) was less than 15,000 NTD, and 63.7% of them belonged to white-collar occupational class. The means of daily concentration of air pollutant exposure were 4.25 (±1.44) ppb for SO_2_, 20.41 (±6.65) ppb for NOx, 9.25 (±4.36) ppb for NO, 20.99 (±3.33) ppb for NO_2_, and 30.85 (±6.16) μg/m^3^ for PM_2.5_.

The distributions of urbanization level among the different quartiles of air pollutants are displayed in Table 2. Most participants exposed to Q4 of SO_2_, NO, and PM_2.5_ lived in urbanization level two area. Participants exposed to Q4 of NOx and NO_2_ mainly lived in urbanization level one area.

The incidence rates and the hazard ratios of PCOS of the five air pollutants are presented in Table 3. The incidence rate of polycystic ovary syndrome was 2.26%. Considering participants exposed to Q1 of air pollutants as the reference group, PCOS risk in participants exposed to Q4 of air pollutants was determined. Relative to level Q1 of SO_2_ concentration, the subjects exposed to level Q4 were associated with a 7.58-fold higher risk of PCOS (aHR = 7.58, 95% CI = 6.18–9.30). In NOx, relative to Q1, the adjusted hazard ratios (HRs) of PCOS risk were 1.58-fold (aHR = 1.58, 95% CI = 1.32–1.88) and 2.99-fold (aHR = 2.99, 95% CI = 2.56–3.50) higher for levels Q3 and Q4, respectively. In NO, compared with Q1, the adjusted HRs of PCOS risk were 3.82-fold (aHR = 3.82, 95% CI = 3.57–4.89) higher for levels Q4. In NO_2_, compared with Q1, the adjusted HRs of PCOS risk were 4.31-fold (aHR = 4.31, 95% CI = 3.72–5.00) for Q4 level. In PM_2.5_, relative to Q1, the adjusted HRs of PCOS risk were 3.34-fold (aHR = 3.34, 95% CI = 2.87–3.89) higher for levels Q4.

Table 4, Table 5, Table 6, Table 7 and Table 8 show the results of stratification analysis. Almost all the patients who were exposed to Q2–Q4 of air pollutants had significantly higher PCOS risk compared with those exposed to Q1 in each stratification of age, urbanization level, and monthly income. 

## 4. Discussion

This is the first epidemiological study to demonstrate that inhalation of increased concentrations of Q4, PM_2.5_, NOx, NO_2_, NO, and SO_2_ in particular is associated with a high PCOS risk. Although our data could not demonstrate the perfect dosage–response relationship between concentrations of air pollutants and PCOS development, these data indicated that air pollution was associated with a high PCOS incidence. We hypothesized several mechanisms that accounted for these findings. First, exposure to fine air pollutants and acid gases might lead to active oxidative stress, mitochondrial damage, DNA damage, lipid peroxidation, and so on, predisposing PCOS development in women, which shares the pathway with non-alcoholic fatty liver disease (NAFLD) [33]. Reports have revealed that NAFLD was prevalent in patients with PCOS, of which the common factors were insulin resistance, hyperandrogenism, and dyslipidemia [34,35,36]. Therefore, PCOS could be viewed as an ovarian component and NAFLD as a liver component of the metabolic syndrome [37]. Mounting evidence indicates that air pollutions are associated with a high incidence of NAFLD and metabolic syndrome [38,39,40,41,42] through the activation of Kupffer cells [43], c-Jun N-terminal Kinases-activator protein 1, nuclear factor-κB, Toll-like receptor-4, and peroxisome proliferator-activated receptors [44]. These activated inflammation processes can lead to increased levels of triglycerides, low-/very low-density lipoproteins, lipid accumulation within hepatocytes, reduced glycogen storage within hepatocytes, and finally insulin resistance and altered carbohydrate metabolism [45,46]. The resultant insulin resistance can predispose vulnerable women to develop PCOS.

Second, air pollution might cause androgen excess through insulin resistance and thus promote PCOS development. Recent studies have shown that air pollution and air particulate matter are closely associated with androgenetic alopecia [47]. Noval et al. [33] showed that air particulate pollutants may exert anti-estrogenic and antiandrogenic effects in vitro. Until now, the direct mechanism of the effect of air particulate pollution on androgen receptors and associated pathways in women, especially those vulnerable to PCOS, was unknown. However, the environmental pollutant BPA was reported to potentially interfere with testosterone synthesis, androgen level, and PCOS development [48]. Although we did not analyze the level of atmospheric BPA in our study, it is possible that BPA adsorbed on these fine air particles led to increased PCOS incidence. Third, PCOS signifies altered endocrine function [49]. Therefore, we speculated that air pollutants would potentially interfere with homeostasis of thyroid hormone as well as the hypothalamus pituitary adrenal axis [50]. Since the functions of gonads were delicately regulated and influenced by the two endocrine axis, dysfunction of gonad would develop inclusive of sex hormone dysregulation and unbalance. Therefore, it might explain the increased incidence of PCOS in those who exposed to higher concentrations of air pollutions [12].

Since our study is the first research investigating the association between PCOS and air pollution, it would be of interest to compare the levels of fine particles in countries other than ours. Cheng et al. reported that annual average concentrations of PM2.5 were highest in Delhi (143.0 ± 17.8 μg/m^3^), Cairo (109.6 ± 27.7 μg/m^3^), and Xi’an (102.2 ± 9.3 μg/m^3^) [51], which were significantly higher than ours. Further, there have been studies showing that Asian cities had more air pollution with the average concentrations of PM_2.5_ and PM_10_ in the cities, respectively, 44–168 and 54–262 μg/m^3^ in the dry season, and 18–104 and 33–180 μg/m^3^ in the wet season [52]. The daily average concentrations of SO_2_ and NO_2_ in Beijing were 8.6 and 25.4 ppb, respectively, which were also both higher than ours. Therefore, similar studies about association between PCOS and air pollutants should be conducted in these seriously air polluted areas in regard to women’s health. 

We also noted that, even after adjusting for air pollutants, white class and higher income levels were associated with higher incidences of PCOS. Merkin et al. found an association between childhood low socioeconomic status and PCOS [53]. They did not find association between higher education or higher socioeconomic status and PCOS [53], as noted in our study. A previous study reported that low socioeconomic status is related with many conditions predisposing one to PCOS, such as insulin resistance [54]. However, we did not come to a similar conclusion. One speculation was that women in white class and higher income levels might have more emotional stress predisposing them to the development of PCOS. Our study results prompt concerns for those women of higher economic status and those who live in more polluted areas. 

This investigational study has several limitations. First, although we considered income and education level, the participants’ actual occupations, personal dietary habits, and daily exercise regimens were unknown. Therefore, whether participants had occupational exposure to environmental hormones, consumed androgen-like foods, or had other behaviors that enhanced insulin resistance for promoting PCOS development or enhanced insulin sensitivity for decreasing the PCOS risk was unknown. Second, information regarding family history of PCOS, body mass index, insulin level, levels of inflammation markers such as homocysteine, sedimentation rate of erythrocytes, C-reactive protein, and so on was unknown. In addition, whether patients had taken over-the-counter medications was unknown. Third, we used ICD-9-CM code of upper respiratory infection (URI), which was the most common disease as a link for the analysis of the TAQMD and NHIRD, to define the location of each participant. The actual mobility of each participant was unknown. Furthermore, information regarding steps taken by participants to protect themselves from air pollutants, such as use of air conditioner or N95 mask, was unknown. Finally, this study did not investigate whether air pollutants had a synergistic effect on PCOS risk.

## 5. Conclusions

Our study showed that increased exposure to fine air pollutant particles and pollutant gases was associated with increased PCOS risk. Our results provide reliable epidemiological information regarding the possible preventable risk factors for PCOS.

## Figures and Tables

**Table 1:** Baseline demographics and exposure of air pollutants in Taiwan.

*N* = 91,803	*n*	%
Age, years		
<20	19,604	21.3
20–64	64,870	70.7
≥65	7329	7.98
Mean, SD ^†^	36.88 (18.78)	
Area		
North	60,294	65.7
Central	9219	10
Southern	22,290	24.3
Urbanization level		
1 (highest)	37,457	40.8
2	47,389	51.6
3	6957	7.58
4 (lowest)	0	0
Monthly income		
<15,000	52,183	56.9
15,000–29,999	30,312	33
≥30,000	9308	10.1
Occupational class		
White color class	58,483	63.7
Blue color class	25,072	27.3
Other	8248	8.98
SO_2_ level (daily average, ppb)		
Mean, SD ^†^	4.25 (1.44)	
Min	2.59	
Lower quartile	2.98	
Median	3.93	
Upper quartile	5.4	
Max	12.27	
NO_x_ level (daily average, ppb)		
Mean, SD ^†^	30.41 (6.65)	
Min	17.81	
Lower quartile	24.39	
Median	30.32	
Upper quartile	34.61	
Max	65.36	
NO level (daily average, ppb)		
Mean, SD ^†^	9.25 (4.36)	
Min	2.92	
Lower quartile	5.47	
Median	7.21	
Upper quartile	12.21	
Max	34.32	
NO_2_ level (daily average, ppb)		
Mean, SD ^†^	20.99 (3.33)	
Min	9.78	
Lower quartile	19.05	
Median	22.31	
Upper quartile	22.95	
Max	31.77	
PM_2.5_ level (daily average, ppb)		
Mean, SD ^†^	30.85 (6.16)	
Min	22.49	
Lower quartile	27.23	
Median	27.41	
Upper quartile	34.78	
Max	67.45	
Outcome		
Polycystic ovaries	2072	2.26
Follow-up time, years (mean, SD) ^†^	7.76 (3.79)	

SO_2_, sulfur dioxide; NOx, nitrogen oxides; NO, nitrogen monoxide; NO_2_, nitrogen dioxide; PM_2.5_, particles with aerodynamic diameter < 2.5 μm; SD, standard deviation. ^†^ Average age, SO_2,_ NOx, NO, NO_2_, PM_2.5_ and follow-up time using Wilcoxon rank-sum test for verification. The urbanization level was categorized by the population density of the residential area into four levels, with level one as the most urbanized and level four as the least urbanized.

**Table 2:** Baseline urbanization level among quartiles of daily average concentration of air pollutants in Taiwan.

Air Pollutant Concentration	Quartile 1 (Q1) (Lowest)		Quartile 2 (Q2)		Quartile 3 (Q3)		Quartile 4 (Q4) (Highest)		*p*-Value
*N* = 91,803	*n*	%	*n*	%	*n*	%	*n*	%	
SO_2_									
Urbanization Level									<0.0001
1 (highest)	17,521	76.3	3251	15.3	10,869	44.6	5816	25	
2	5403	23.5	13,754	64.8	10,937	44.9	17,295	74.4	
3	25	0.11	4229	19.9	2571	10.5	132	0.57	
4 (lowest)	0	0	0	0	0	0	0	0	
NOx									
Urbanization Level									<0.0001
1 (highest)	2728	17.2	5541	23.9	10,183	48.9	19,005	59.5	
2	11,483	72.5	15,023	64.8	10,614	50.9	10,269	32.1	
3	1637	10.3	2632	11.3	41	0.2	2647	8.29	
4 (lowest)	0	0	0	0	0	0	0	0	
NO									
Urbanization Level									<0.0001
1 (highest)	8065	46.4	219	1.01	23,414	81.4	5759	23.9	
2	7652	44.1	18,783	86.8	5283	18.4	15,671	65.1	
3	1637	9.43	2623	12.1	49	0.17	2648	11	
4 (lowest)	0	0	0	0	0	0	0	0	
NO_2_									
Urbanization Level									<0.0001
1 (highest)	2719	12.3	10,070	37.3	0	0	24,668	71.8	
2	17,656	80.2	14,309	52.9	8427	100	6997	20.4	
3	1649	7.49	2629	9.73	0	0	2679	7.8	
4 (lowest)	0	0	0	0	0	0	0	0	
PM_2.5_									
Urbanization Level									<0.0001
1 (highest)	4725	22.2	12,963	70.3	11,241	40	8528	35.6	
2	16,561	77.7	5471	29.7	12,706	45.2	12,651	52.8	
3	13	0.06	0	0	4159	14.8	2785	11.6	
4 (lowest)	0	0	0	0	0	0	0	0	

*p*-value using chi-square for the comparisons between urbanization levels among quartiles of daily average concentration of air pollutants. The urbanization level was categorized by the population density of the residential area into four levels, with level one as the most urbanized and level four as the least urbanized. The daily average air pollutant concentrations were categorized based on quartiles for each air pollutants.

**Table 3:** Difference in polycystic ovaries incidences and associated HRs in participant exposed to various daily average concentration of air pollutants.

Pollutant Levels	*N*	Polycystic Ovaries	PY	IR	Crude HR	Adjusted HR
		Event			(95% CI)	(95% CI)
**SO_2_ (Daily Average)**						
Q1	22,949	150	182,211	0.82	1 (reference)	1 (reference)
Q2	21,234	515	165,975	3.1	5.38 (4.36–6.64) ***	7.35 (5.90–9.15) ***
Q3	24,377	676	187,153	3.61	6.27 (5.10–7.70) ***	7.68 (6.23–9.47) ***
Q4	23,243	776	177,701	4.36	7.58 (6.18–9.30) ***	10.31 (8.35–12.7) ***
NOx (Daily Average)						
Q1	15,848	183	125,013	1.46	1 (reference)	1 (reference)
Q2	23,196	449	182,324	2.46	1.68 (1.41–1.99) ***	1.66 (1.39–1.98) ***
Q3	20,838	379	163,543	2.31	1.58 (1.32–1.88) ***	1.66 (1.39–1.99) ***
Q4	31,921	1061	242,160	4.38	2.99 (2.56–3.50) ***	3.37 (2.86–3.96) ***
NO (Daily Average)						
Q1	17,354	193	136,516	1.41	1 (reference)	1 (reference)
Q2	21,625	410	170,528	2.4	1.70 (1.43–2.01) ***	2.03 (1.69–2.44) ***
Q3	28,746	488	224,425	2.17	1.53 (1.30–1.81) ***	1.33 (1.12–1.58) ***
Q4	24,078	981	181,571	5.4	3.82 (3.28–4.46) ***	4.18 (3.57–4.89) ***
NO_2_ (Daily Average)						
Q1	22,024	206	174,601	1.17	1 (reference)	1 (reference)
Q2	27,008	487	212,605	2.29	1.94 (1.65–2.28) ***	2.39 (2.03–2.82) ***
Q3	8427	63	669,83	0.94	0.79 (0.60–1.05)	0.70 (0.53–0.93) *
Q4	34,344	1316	258,851	5.08	4.31 (3.72–5.00) ***	7.46 (6.38–8.71) ***
PM_2.5_ (Daily Average)						
Q1	21,299	214	167,328	1.27	1 (reference)	1 (reference)
Q2	18,434	54	145,796	0.37	0.29 (0.21–0.39) ***	0.26 (0.19–0.36) ***
Q3	28,106	1020	216,470	4.71	3.68 (3.18–4.27) ***	3.94 (3.39–4.58) ***
Q4	23,964	784	183,446	4.27	3.34 (2.87–3.89) ***	3.56 (3.05–4.15) ***

Q1: first quartile; Q2: second quartile; Q3: third quartile; Q4: fourth quartile; PY, person-years; IR, incidence rate, per 1000 person-years; HR, hazard ratio; CI, confidence interval; HR adjusted for gender, age, monthly income, urbanization level, and occupational class. *: *p* < 0.05; ***: *p* < 0.001.

**Table 4:** Incidence rate and hazard ratio of polycystic ovaries between various daily average concentrations of SO_2_ stratified by gender, age and comorbidities.

	SO_2_			
	Adjusted HR (95% CI)			
	Quartile1 (Lowest)	Quartile 2	Quartile 3	Quartile 4 (Highest)
Age, Years				
<20	1 (reference)	6.83 (4.22–11.1) ***	8.69 (5.49–13.7) ***	11.3 (7.13–18.0) ***
20–64	1 (reference)	7.63 (5.88–9.90) ***	7.56 (5.90–9.68) ***	10.2 (8.00–13.1) ***
≥65	1 (reference)	6.80 (3.10–14.9) ***	5.82 (2.71–12.4) ***	7.75 (3.58–16.7) ***
Urbanization Level	1 (reference)			
1 (highest)	1 (reference)	20.4 (15.4–27.2) ***	12.6 (9.59–16.5) ***	10.6 (7.99–14.2) ***
2	1 (reference)	3.31 (2.28–4.81) ***	4.13 (2.84–6.00) ***	5.76 (4.01–8.28) ***
3	1 (reference)	0.01 (0.05–0.01) ***	0.01 (0.007–0.02) ***	0.64 (0.33–1.25)
4 (lowest)	1 (reference)	--	--	--
Monthly Income	1 (reference)			
<15,000	1 (reference)	6.45 (4.73–8.78) ***	8.54 (6.35–11.4) ***	10.4 (7.72–14.0) ***
15,000–29,999	1 (reference)	7.39 (5.10–10.7)8 **	5.05 (3.55–7.18) ***	8.61 (6.05–12.2) ***
≥30,000	1 (reference)	13.9 (7.82–24.8) ***	12.1 (6.98–21.0) ***	13.8 (7.81–24.6)
Occupational Class	1 (reference)			
White color class	1 (reference)	8.50 (6.60–10.9) ***	9.21 (7.23–11.7) ***	10.8 (8.53–13.9) ***
Blue color class	1 (reference)	5.96 (3.62–9.80) ***	4.40 (2.76–7.00) ***	6.54 (4.13–10.3) ***
Other	1 (reference)	12.0 (3.69–39.2) ***	19.8 (6.20–63.4) ***	26.4 (8.30–84.5) ***

HR, hazard ratio; HR adjusted for gender, age, monthly income, urbanization level, and occupational class. **: *p* <0.01; ***: *p* < 0.001.

**Table 5:** Incidence rate and hazard ratio of polycystic ovaries between various daily average concentrations of NOx stratified by gender, age and comorbidities.

	NOx			
	Adjusted HR (95% CI)			
	Quartile1 (Lowest)	Quartile 2	Quartile 3	Quartile 4 (Highest)
Age, Years				
<20	1 (reference)	1.79 (1.26–2.53) ***	1.89 (1.33–2.70) ***	3.68 (2.67–5.07) ***
20–64	1 (reference)	1.61 (1.30–2.00) ***	1.56 (1.25–1.95) ***	3.32 (2.73–4.04) ***
≥65	1 (reference)	1.57 (0.77–3.19)	1.77 (0.87–3.61)	2.86 (1.49–5.50) **
Urbanization Level	1 (reference)			
1 (highest)	1 (reference)	0.74 (0.52–1.05)	0.76 (0.56–1.05)	1.60 (1.20–2.13) **
2	1 (reference)	2.52 (2.00–3.19) ***	2.20 (1.73–2.81) ***	5.42 (4.36–6.73) ***
3	1 (reference)	0.89 (0.56–1.43)	5.69 (2.90–10.6) ***	1.30 (0.83–2.04)
4 (low)	1 (reference)	-	-	-
Monthly Income	1 (reference)			
<15,000	1 (reference)	1.71 (1.38–2.13) ***	1.65 (1.31–2.06) ***	3.08 (2.53–3.67) ***
15,000–29,999	1 (reference)	1.56 (1.10–2.21) *	1.64 (1.15–2.34) **	3.81 (2.75–5.28) ***
≥30,000	1 (reference)	1.69 (0.88–3.23)	1.85 (0.96–3.55)	4.19 (2.34–7.50) ***
Occupational Class	1 (reference)			
White color class	1 (reference)	2.28 (1.85–2.83) ***	1.70 (1.36–2.13) ***	3.45 (2.83–4.20) ***
Blue color class	1 (reference)	0.78 (0.52–1.19)	1.14 (0.75–1.72)	3.02 (2.03–4.50) ***
Other	1 (reference)	2.08 (1.29–3.36) **	1.96 (1.18–3.24) **	2.97 (1.91–4.60) ***

HR, hazard ratio; HR adjusted for gender, age, monthly income, urbanization level, and occupational class. *: *p* < 0.05; **: *p* < 0.01; ***: *p* < 0.001.

**Table 6:** Incidence rate and hazard ratio of polycystic ovaries between various daily average concentrations of NO stratified by gender, age and comorbidities.

	NO			
	Adjusted HR (95% CI)			
	Quartile1 (Lowest)	Quartile 2	Quartile 3	Quartile 4 (Highest)
Age, Years				
<20	1 (reference)	2.09 (1.43–3.03) ***	1.80 (1.26–2.56) **	4.52 (3.26–6.27) ***
20–64	1 (reference)	2.09 (1.67–2.60) ***	1.19 (0.97–1.46)	4.24 (3.51–5.12) ***
≥65	1 (reference)	1.54 (0.76–3.11)	1.45 (0.75–2.76)	2.99 (1.63–5.48) ***
Urbanization Level	1 (reference)			
1 (highest)	1 (reference)	8.07 (5.78–11.2) ***	1.57 (1.19–2.07) **	10.4 (8.01–13.7) ***
2	1 (reference)	1.09 (0.86–1.37)	2.68 (2.10–3.43) ***	2.44 (1.97–3.03) ***
3	1 (reference)	0.78 (0.48–1.26)	9.06 (4.66–17.6) ***	1.44 (0.92–2.24)
4 (low)	1 (reference)	-	-	-
Monthly Income	1 (reference)			
<15,000	1 (reference)	1.92 (1.53–2.41) ***	1.31 (1.05–1.62) *	3.70 (3.05–4.49) ***
15,000–29,999	1 (reference)	2.23 (1.57–3.17) ***	1.62 (1.17–2.25) **	4.66 (3.41–6.35) ***
≥30,000	1 (reference)	2.50 (1.25–5.01) **	1.02 (0.54–1.94)	6.94 (3.81–12.6) ***
Occupational Class	1 (reference)			
White color class	1 (reference)	2.22 (1.77–2.78) ***	1.04 (0.84–1.29)	4.24 (3.52–5.11) ***
Blue color class	1 (reference)	1.82 (1.14–2.90) *	2.18 (1.47–3.25) ***	4.56 (2.95–7.05) ***
Other	1 (reference)	2.09 (1.26–3.46) **	2.13 (1.31–3.46) **	3.00 (1.97–4.59) ***

HR, hazard ratio; HR adjusted for gender, age, monthly income, urbanization level, and occupational class. *: *p* < 0.05; **: *p* < 0.01; ***: *p* <0.001.

**Table 7:** Incidence rate and hazard ratio of polycystic ovaries between various daily average concentrations of NO_2_ stratified by gender, age and comorbidities.

	NO_2_			
	Adjusted HR (95% CI)			
	Quartile1 (Lowest)	Quartile 2	Quartile 3	Quartile 4 (Highest)
Age, Years				
<20	1 (reference)	2.32 (1.67–3.22) ***	0.99 (0.61–1.62)	7.76 (5.73–10.5) ***
20–64	1 (reference)	2.41 (1.97–2.94) ***	0.57 (0.39–0.82) **	7.52 (6.22–9.08) ***
≥65	1 (reference)	2.59 (1.31–5.12) **	1.08 (0.37–3.13)	6.56 (3.39–12.7) ***
Urbanization Level				
1 (highest)	1 (reference)	0.46 (0.33–0.65) ***	1.53 (1.16–2.03) **	0.99 (0.99–1.00)
2	1 (reference)	3.70 (3.00–4.57) ***	1.09 (0.80–1.49)	13.2 (10.8–16.0) ***
3	1 (reference)	0.75 (0.48–1.19)	-	1.34 (0.88–2.04)
4 (low)	1 (reference)	-	-	-
Monthly Income				
<15,000	1 (reference)	2.13 (1.73–2.62) ***	0.72 (0.51–1.02)	6.35 (5.24–7.71) ***
15,000–29,999	1 (reference)	2.98 (2.17–4.08) ***	0.63 (0.35–1.15)	9.43 (6.95–12.8) ***
≥30,000	1 (reference)	2.65 (1.47–4.80) **	0.78 (0.32–1.92)	10.8 (6.23–18.8) ***
Occupational Class				
White color class	1 (reference)	2.80 (2.29–3.43) ***	0.52 (0.36–0.76) ***	6.97 (5.77–8.43) ***
Blue color class	1 (reference)	2.38 (1.66–3.42) ***	1.68 (0.96–2.93)	11.9 (8.39–17.0) ***
Other	1 (reference)	1.45 (0.90–2.35)	0.80 (0.36–1.76)	5.32 (3.45–8.21) ***

HR, hazard ratio; HR adjusted for gender, age, monthly income, urbanization level, and occupational class. **: *p* < 0.01; ***: *p* < 0.001.

**Table 8:** Incidence rate and hazard ratio of polycystic ovaries between various daily average concentrations of PM_2.5_ stratified by gender, age and comorbidities.

	PM_2.5_			
	Adjusted HR (95% CI)			
	Quartile1 (Lowest)	Quartile 2	Quartile 3	Quartile 4 (Highest)
Age, Years				
<20	1 (reference)	0.14 (0.06–0.33) ***	4.07 (3.01–5.52) ***	3.95 (2.90–5.39) ***
20–64	1 (reference)	0.30 (0.21–0.42) ***	3.99 (3.33–4.78) ***	3.45 (2.87–4.15) ***
≥65	1 (reference)	0.23 (0.06–0.82) *	3.14 (1.75–5.63) ***	3.35 (1.85–6.08) ***
Urbanization Level				
1 (highest)	1 (reference)	0.20 (0.13–0.30) ***	3.95 (3.05–5.13) ***	2.18 (1.66–2.86) ***
2	1 (reference)	0.29 (0.16–0.50) ***	4.37 (3.62–5.26) ***	4.26 (3.53–5.14) ***
3	1 (reference)	0.01 (0.003–0.03) ***	0.02 (0.008–0.08) ***	0.99 (0.98–1.01)
4 (low)	1 (reference)	-	-	-
Monthly Income				
<15,000	1 (reference)	0.18 (0.11–0.29) ***	3.87 (3.16–4.73) ***	3.67 (2.99–4.51) ***
15,000–29,999	1 (reference)	0.48 (0.30–0.75) **	3.91 (3.00–5.09) ***	3.52 (2.69–4.60) ***
≥30,000	1 (reference)	0.18 (0.08–0.40) ***	4.51 (2.94–6.92) ***	3.02 (1.91–4.77) ***
Occupational Class				
White color class	1 (reference)	0.27 (0.19–0.39) ***	5.17 (4.24–6.29) ***	4.49 (3.66–5.50) ***
Blue color class	1 (reference)	0.40 (0.22–0.72) **	2.68 (2.04–3.52) ***	2.15 (1.65–2.80) ***
Other	1 (reference)	-	3.47 (2.08–5.79) ***	3.40 (2.04–5.65) ***

HR, hazard ratio; HR adjusted for gender, age, monthly income, urbanization level, and occupational class. *: *p* < 0.05; **: *p* < 0.01; ***: *p* < 0.001.

## Supplementary Materials

The following are available online at https://www.mdpi.com/1660-4601/16/23/4816/s1, Figure S1: The map of study area, Figure S2: Air quality monitoring site map, Figure S3: Annual average concentration distribution of SO2 in 1999 to 2013, Figure S4: Annual average concentration distribution of NOx in 1999 to 2013, Figure S5: Annual average concentration distribution of NO in 1999 to 2013, Figure S6: Annual average concentration distribution of NO2 in 1999 to 2013, Figure S7: Annual average concentration distribution of PM2.5 in 1999 to 2013.
