# Supplementary material for: Risk of Polycystic Ovary Syndrome in Women Exposed to Fine Air Pollutants and Acidic Gases: A Nationwide Cohort Analysis
Source: Int J Environ Res Public Health. 2019 Nov 30;16(23):4816. doi: 10.3390/ijerph16234816 (PMC6926786; doi:10.3390/ijerph16234816)
Supplement: Supplementary file 1 [file ijerph-16-04816-s001.pdf]

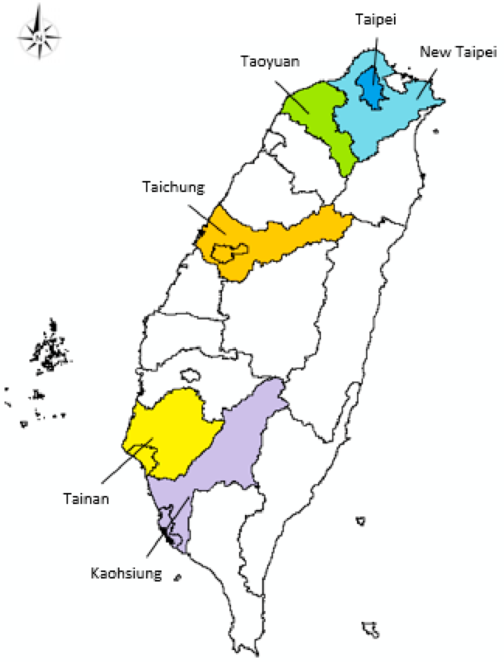

Supplementary Figure S1. The map of study area.

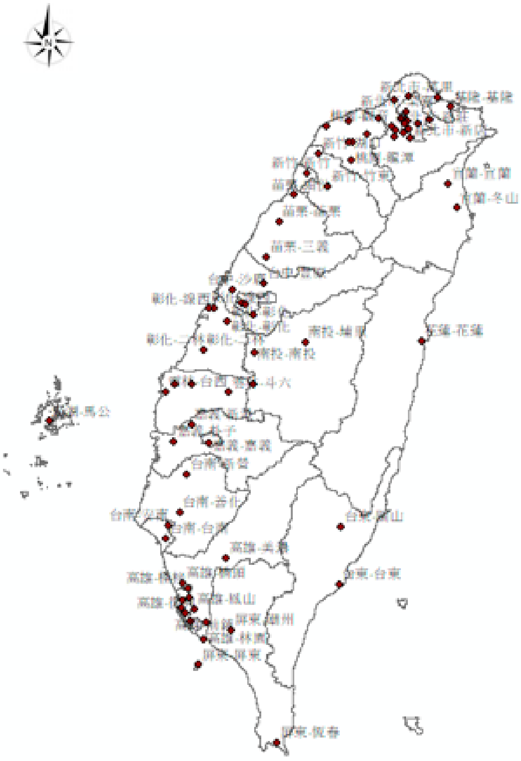

Supplementary Figure S2. Air quality monitoring site map.

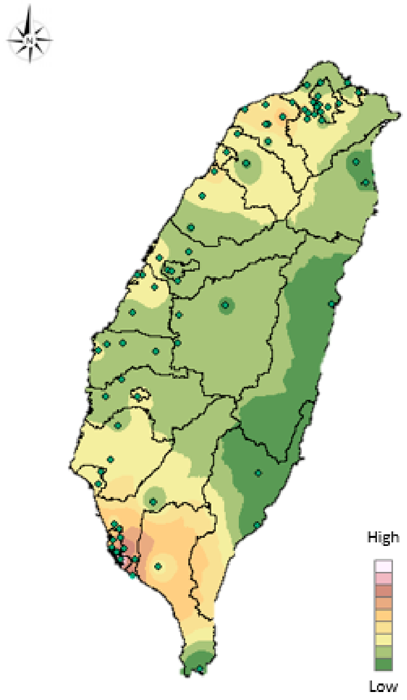

Supplementary Figure S3. Annual average concentration distribution of SO_2 in 1999 to 2013.

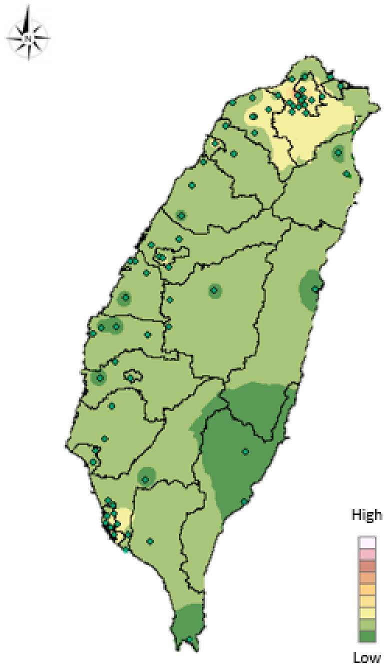

Supplementary Figure S4. Annual average concentration distribution of NOx in 1999 to 2013.

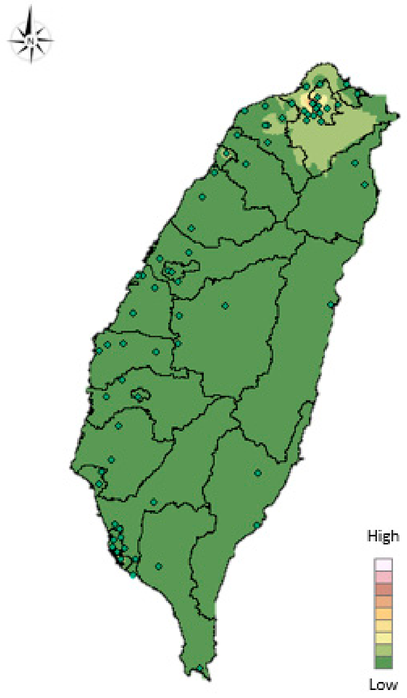

Supplementary Figure S5. Annual average concentration distribution of NO in 1999 to 2013.

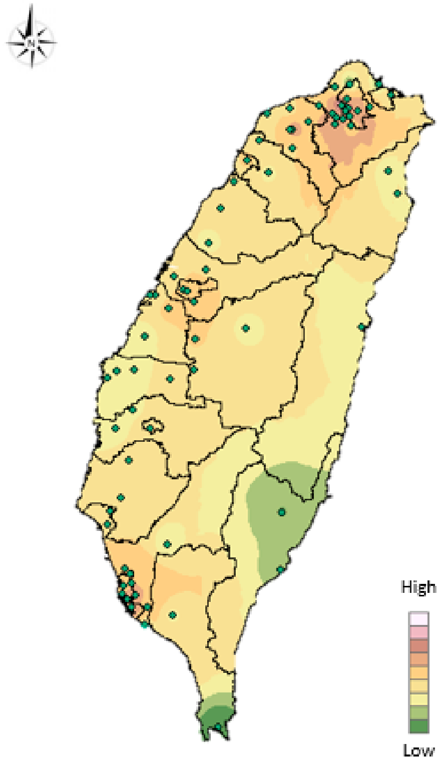

Supplementary Figure S6. Annual average concentration distribution of NO_2 in 1999 to 2013.

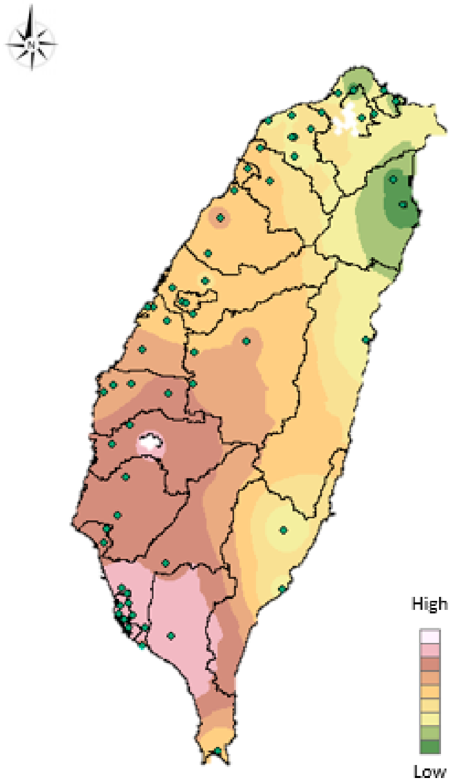

Supplementary Figure S7. Annual average concentration distribution of $PM_{2.5}$ in 1999 to 2013.
